# Interactive three-dimensional atlas of the mineralized skeleton of the sand tiger shark *Carcharias taurus*

**DOI:** 10.1098/rsos.240287

**Published:** 2024-05-01

**Authors:** Emil Alexander Byriel Winkel, Rune Kristiansen, Peter Rask Møller, Henrik Lauridsen

**Affiliations:** ^1^ Department of Clinical Medicine, Aarhus University, Aarhus N 8200, Denmark; ^2^ Department of Forensic Medicine, Aarhus University, Aarhus N 8200, Denmark; ^3^ Kattegatcentret, Grenaa, 8500, Denmark; ^4^ Natural History Museum of Denmark, University of Copenhagen, København Ø, Denmark

**Keywords:** computed tomography, anatomy, model, elasmobranch, Selachimorpha

## Abstract

Non-invasive computed tomography (CT) of an adult sand tiger shark *Carcharias taurus* Rafinesque, 1810 is used to provide an interactive three-dimensional ‘general’ shark (Selachimorpha) anatomy atlas. Given its post-cranial body morphology, the sand tiger shark appeared to be a well-chosen candidate and through comparison of the sand tiger shark with several other representatives of all eight established orders of sharks, we confirm that the relatively large degree of mineralization of the endoskeleton, along with the overall size, makes the sand tiger shark an ideal candidate for skeletal segmentation and construction of a skeletal atlas using conventional CT. This atlas both increases accessibility to the internal morphological features of the sand tiger shark and provides a more generalized overview of the skeletal anatomy of sharks and can aid as a supplement to destructive fresh dissection of specimens in the future and the construction of future skeletal atlases of other less mineralized sharks.

## 1. Introduction

Traditionally, the study of internal anatomy in sharks (Selachimorpha) has relied heavily on invasive dissections of specimens, subsequently resulting in the generation of realistic two-dimensional illustrations [1–5]. This practice relies heavily on skilful anatomists, talented illustrators and, above all else, a tremendous amount of time. In more recent times, non-invasive imaging modalities such as X-ray computed tomography (CT) have become common methods for investigating internal anatomy in medical practices [6]. Similarly, CT can be applied to zoological specimens to rapidly generate three-dimensional representations of dense anatomical structures in detail that are able to rival that of more traditional anatomical illustrations based on dissection and allow for various quantitative measurements to be obtained [7–9].

In addition to creating still images of the specimens, the use of CT allows for the creation of interactive three-dimensional models of the anatomy. Modalities such as CT have been used extensively to describe human anatomy [10,11] and generate web-based libraries containing anatomical data of a range of animal species [12]. These practices have been applied extensively to model and illustrate the anatomy of classic experimental animals such as mice for some time now [13,14]. In recent times, these techniques have made their way into the fields of comparative anatomy and organismal biology, and rapid advances in visualization techniques [15] have created a boom in digitalized anatomical studies [16–19].

CT has been used to study shark anatomy for some time. Many studies, however, focused on species-specific, detailed anatomical features [20–25], rather than larger aspects of full-body morphology. Sharks’ skeletons consist mostly of cartilage covered by a thin layer of mineralized tiles called tesserae [26]. The arrangements and morphologies of mineralized skeletons vary greatly with species, age, location in the body as well as a number of ecological factors, such as individual/population feeding behaviour [27,28]. The degree of mineralization ultimately determines if a given skeletal structure will be distinguishable from the surrounding soft tissues on the CT scans, making it possible to segment it from the initial image data.

Sharks have evolved a number of different feeding mechanisms ranging from filter-feeding, as seen in Cetorhinidae, Megachasmidae and Rhincodontidae, to cutting/tearing in the Carcharhiniformes and Lamniformes as well as crushing in the Orectolobiformes and Heterodontiformes, which, in turn, has given rise to a large diversity in cranial morphology [29].

**Figure 1.**
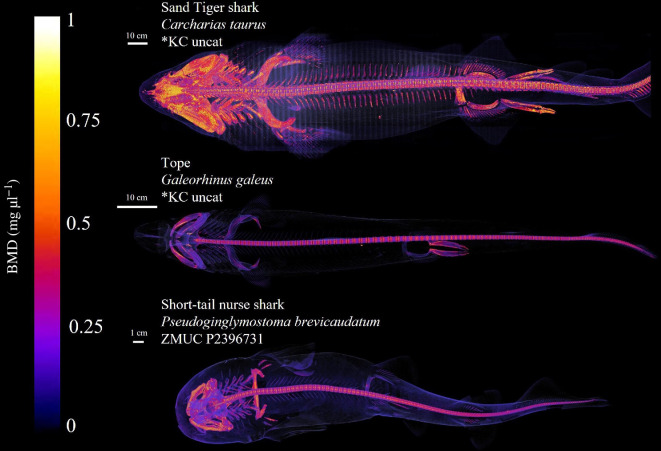
Volume rendering of bone mineral density (BMD) map of the scanned sand tiger shark specimen’s endoskeleton along with other somewhat well-mineralized specimens. Note the larger degree of mineralization in the sand tiger shark around the branchial arches, the pectoral fin radials and the pelvic fin radials.

With more than 500 species and more than 500 Myr of evolution [30], large generalizations can be difficult to make regarding the morphology of the internal anatomy of sharks. The sand tiger shark belongs to the Galeomorphii subclass and the order Lamniformes but does not represent a basal lineage in either of them [31]. Preliminary studies, however, showed that this is a very well-ossified species, and thus a suitable reference model for studies in shark osteology and imaging-based physiology (figure 1). This, along with the overall post-cranial morphology [32–34], makes the sand tiger shark likely to serve as a well-suited candidate for a more general anatomical model, with the caveat that one naturally cannot reduce the diverse morphology of sharks to a single species. Several features of the sand tiger shark morphology make it a valid candidate, namely having a clear and well-defined presence of all paired and unpaired fin structures, as well as an overall lack of rare structural adaptations, that is a dorsoventrally flattened body like the Squatiniformes and Orectolobiformes, or extreme morphological adaptations to the cranial region as in the Pristioforiformes, Sphyrnidae or *Mitsukurina owstoni*. It possesses a relatively deep body, but is more ventrally flattened than the really deep-bodied species, that is Lamnidae, a rather conical head, but more ventrally flattened than other Lamniformes, more reminiscent of the typical Carcharhinidae head profile, and finally, a moderate heterocercal angle and aspect ratio of the caudal fin [35]. All these morphological features make the sand tiger shark a well-suited exemplary representative of many aspects of shark morphology [32–34].

Other studies have pursued similar endeavours of providing anatomical studies aided by modern imaging techniques [36,37], but, to our knowledge, none of these have included three-dimensional or otherwise interactive representations. One noteworthy online resource for chondrichthyan anatomy is the website ‘Chondrichthyan Tree of Life’ [38] which, among other things, offers an impressive selection of less in-depth segmented anatomical models than the one presented in this article.

The aim of the present study was to provide an interactive three-dimensional atlas for the sand tiger shark. We expect that the relatively large degree of mineralization of the endoskeleton, along with the overall size, makes the sand tiger shark an ideal model for skeletal segmentation and constructions of future skeletal atlases of other less mineralized sharks and educational purposes. We compare the sand tiger shark with representatives from all eight orders of Selachimorpha (table 1) in order to confirm that the sand tiger shark is indeed among the most mineralized sharks and as such a suitable candidate for skeletal segmentation using conventional clinical CT.

**Table 1.** List of included species of sharks from all eight orders.

order	family	species	catalogue #
Carcharhiniformes	Carcharhinidae	*Prionace glauca*	ZMUC P0577
Carcharhiniformes	Scyliorhinidae	*Scyliorhinus canicula*	^a^KC uncat.
Carcharhiniformes	Sphyrnidae	*Sphyrna tiburo*	^a^KC uncat.
Carcharhiniformes	Triakidae	*Galeorhinus galeus*	^a^KC uncat.
Heterodontiformes	Heterodontidae	*Heterodontus portusjacksoni*	^a^KC uncat.
Heterodontiformes	Heterodontidae	*Heterodontus ramalheira*	ZMUC P0598
Hexanchiformes	Chlamydoselachidae	*Chlamydoselachus anguine*	ZMUC 1
Hexanchiformes	Hexanchidae	*Hexanchus griseus*	ZMUC P0564
Lamniformes	Alopiidae	*Alopias vulpinus*	ZMUC CN5
Lamniformes	Cetorhinidae	*Cetorhinus maximus*	ZMUC P0571
Lamniformes	Lamnidae	*Isurus oxyrinchus*	ZMUC P2394710
Lamniformes	Lamnidae	*Lamna nasus*	ZMUC P2397040
Lamniformes	Mitsukurinidae	*Mitsukurina owstoni*	ZMUC CN1
Lamniformes	Odontaspididae	*Carcharias taurus*	^a^KC uncat.
Lamniformes	Pseudocarchariidae	*Pseudocarcharias kamoharai*	ZMUC 7
Orectolobiformes	Ginglymostomatidae	*Ginglymostoma cirratum*	ZMUC 50
Orectolobiformes	Ginglymostomatidae	*Pseudoginglymostoma brevicaudatum*	ZMUC P2396731
Orectolobiformes	Hemiscylliidae	*Chiloscyllium arabicum*	ZMUC CN2
Orectolobiformes	Hemiscylliidae	*Chiloscyllium grisium*	ZMUC P2395649
Orectolobiformes	Hemiscylliidae	*Chiloscyllium indicum*	ZMUC 78
Orectolobiformes	Hemiscylliidae	*Chiloscyllium plagiosum*	ZMUC 38
Orectolobiformes	Hemiscylliidae	*Chiloscyllium punctatum*	ZMUC P2395650
Orectolobiformes	Hemiscylliidae	*Hemiscyllium ocellatum*	ZMUC 425
Orectolobiformes	Orectolobidae	*Orectolobus japonicus*	ZMUC CN1
Orectolobiformes	Rhincodontidae	*Rhincodon typus*	MNHN2144
Orectolobiformes	Stegostomatidae	*Stegostoma tigrinum*	^a^KC uncat.
Pristioforiformes	Pristiophoridae	*Pristiphorus japonicus*	ZMUC a
Squaliformes	Centrophoridae	*Centrophorus squamosus*	ZMUC 4
Squaliformes	Somniosidae	*Centroscymnus coelolepis*	ZMUC P07165
Squaliformes	Somniosidae	*Somniosus microcephalus*	ZMUC P07159
Squaliformes	Squalidae	*Squalus acanthias*	ZMUC P07193
Squatiniformes	Squatinidae	*Squatina oculata*	ZMUC P0586
Squatiniformes	Squatinidae	*Squatina squatina*	ZMUC V

^a^ KC uncat. are uncatalogued species from Kattegatcentret.

## 2. Material and methods

### 2.1. Specimens

The detailed interactive anatomical atlas was created from a CT scan of an euthanized adult (greater than 30 years old) sand tiger shark from the public Danish aquarium, Kattegatcentret, originally captured in Florida Keys, USA, in 1993. Additionally, specimens from another 32 species (spanning 8 orders and 22 families) were CT imaged to provide information about the anatomical variation of the mineralized skeleton across a wide range of sharks (table 1). Most specimens were from the collection at the Natural History Museum of Denmark and are currently preserved in 70% v/v ethanol. Additional specimens were obtained from the public aquarium Kattegatcentret and were scanned in either a frozen or thawed state.

### 2.2. Imaging

Quantitative X-ray CT was performed using either a Siemens Somatom Definition Dual Energy system or a Toshiba Aquillon Prime SP system with the following parameters: X-ray tube voltage = 120 kVp, X-ray tube current = 260–450 mA, integration time = 1000 ms, spatial resolution = 0.4–1.0 mm isotropic, convolution kernel = B45 s (Siemens) and FC18 (Toshiba), acquisition time = 60–90 s per scan. Underneath the specimens, a Mindways QCT Pro bone mineral calibration phantom was positioned to calibrate X-ray attenuation values to bone mineral density (mg mm^−3^ equivalent aqueous K_2_HPO_4_). The sand tiger shark specimen was imaged in its entirety except for the caudal fin using both a low-resolution full body scan and a high-resolution tile scan spanning small portions of the specimen that were subsequently combined using ImageJ 1.50e creating a combined high-resolution dataset.

Subsequently, the head region was imaged at high resolution with a separate tile scan and a low-resolution scan with the mouth open using a pillow as support.

### 2.3. Segmentation and interactive model construction

The segmentation of the various skeletal components was performed using the visual imaging software Amira version 5.3.3, in which isosurfaces were created using various thresholds. From this, the EXTRACTSURFACE module was applied along with the SURFACEVIEW module. Afterwards, the SURFACE EDITOR tool was used to create surface models of the various anatomical components, which were exported in Wavefront format (.obj). The model construction was performed using the Adobe Acrobat 3D version 8 Toolkit and assembled in the Universal 3D format (.u3d). Afterwards, Interactive PDF files of each model were generated in Adobe Acrobat 3D version 8. Anatomical visualizations of the mineralized skeleton of other shark specimens were performed in Blender version 3.6.1 LTS.

## 3. Results

**Figure 2.**
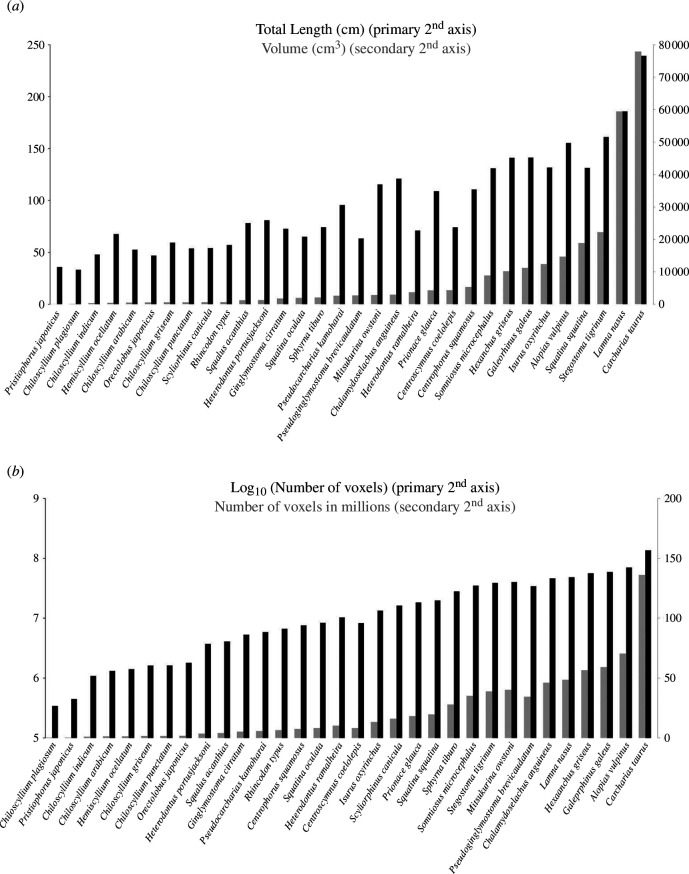
(*a*) Size of imaged shark specimens indicated as total length (in black) on primary second axis and full body volume (in grey) on secondary second axis. (*b*) Image resolution in a selection of imaged shark species indicated as average number of voxels (total in grey and log10-transformed in black). Secondary second axis numbers are given in millions.

**Figure 3.**
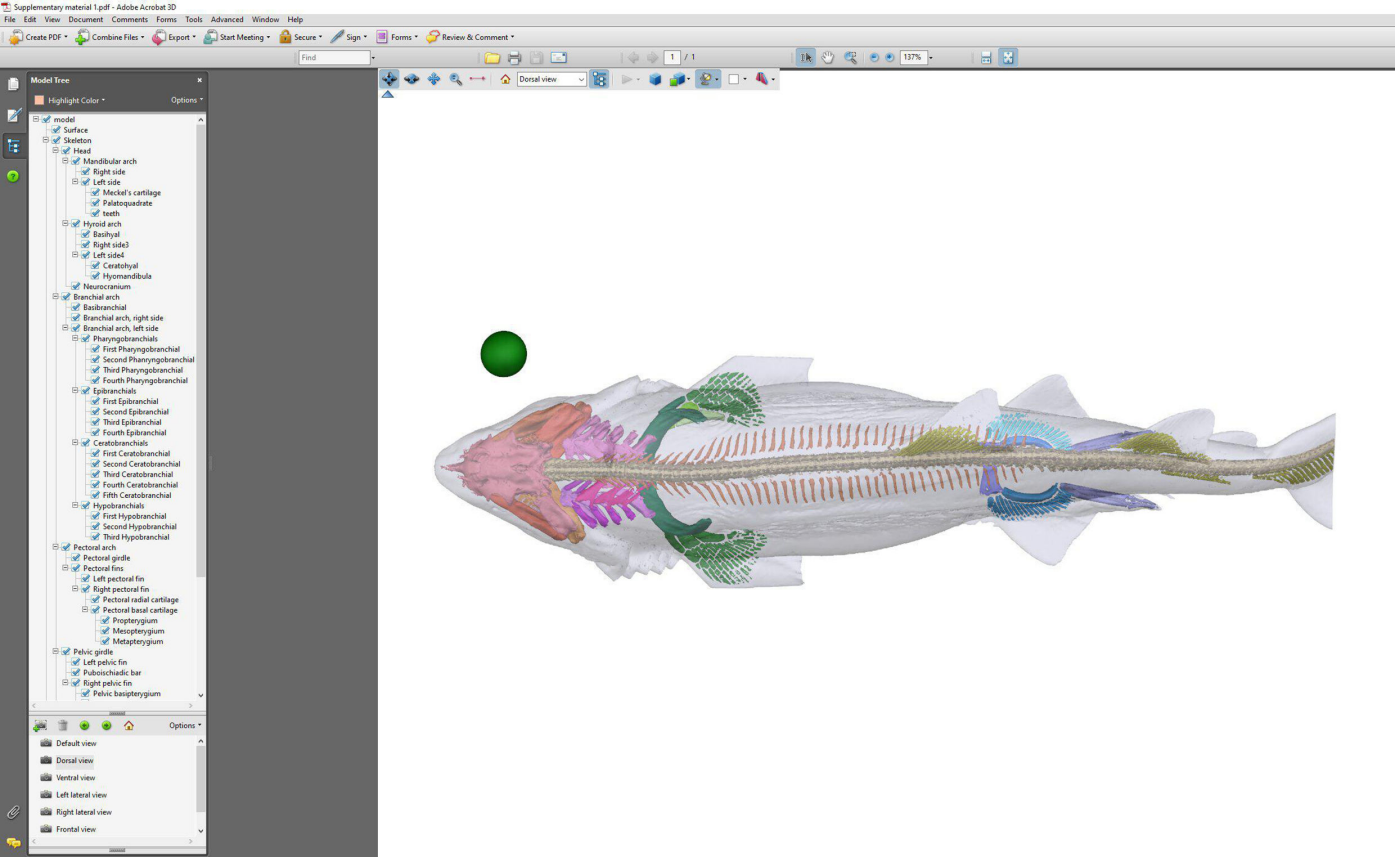
Screenshot of the interactive three-dimensional atlas of the mineralized skeleton of the sand tiger shark. The name of each anatomic structure along with its anatomical association is displayed in the tab to the left. From here, each structure can be selected and deselected for display at a given time while retaining spatial orientation.

**Figure 4.**
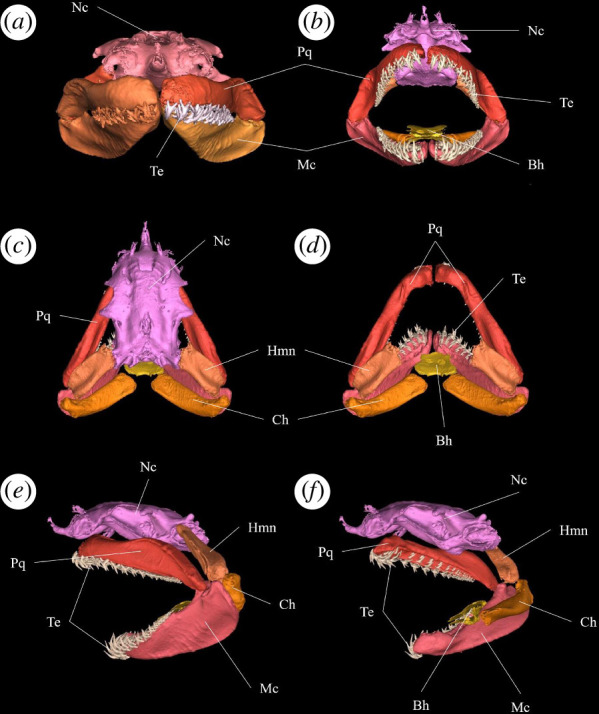
Sand tiger shark (*KC uncat), various views of a skeletal model of the head. (*a*) Anterior view of the head, jaws closed; (*b*) anterior view of head, jaws open; (*c*) dorsal view of the head; (*d*) dorsal view of the head, neurocranium removed; (*e*) left lateral view of the head, jaws open; (*f*) left lateral view of the head, jaws open, left side of mandibular arch and hyoid arch removed. Anatomical abbreviations: Bh, basihyal; Ch, ceratohyal; Hmn, hyomandibula; Mc, Meckel’s cartilage; Nc, neurocranium; Pq, palatoquadrate; Te, teeth.

**Figure 5.**
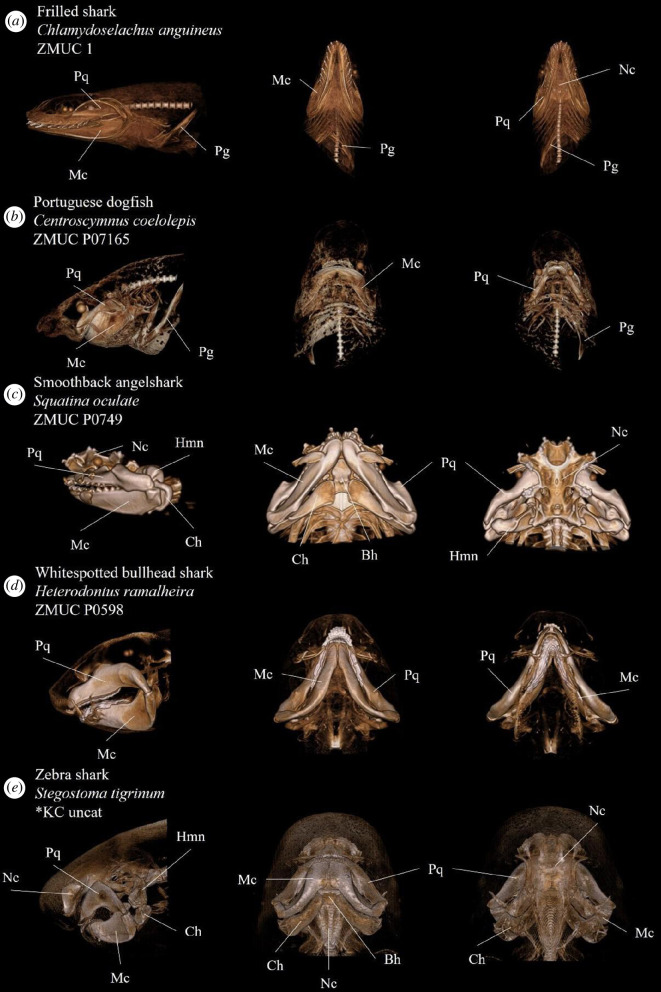
Annotated volume renderings depicting morphological variation of the elasmobranch cranial anatomy in various species. Rows display different views of the same specimens. From left to right; left lateral view, ventral view and dorsal view. Anatomical abbreviations: Bh, basihyal; Ch, ceratohyal; Hmn, hyomandibula; Mc, Meckel’s cartilage; Nc, neurocranium; Pg, Pectoral girdle; Pq, palatoquadrate.

**Figure 6.**
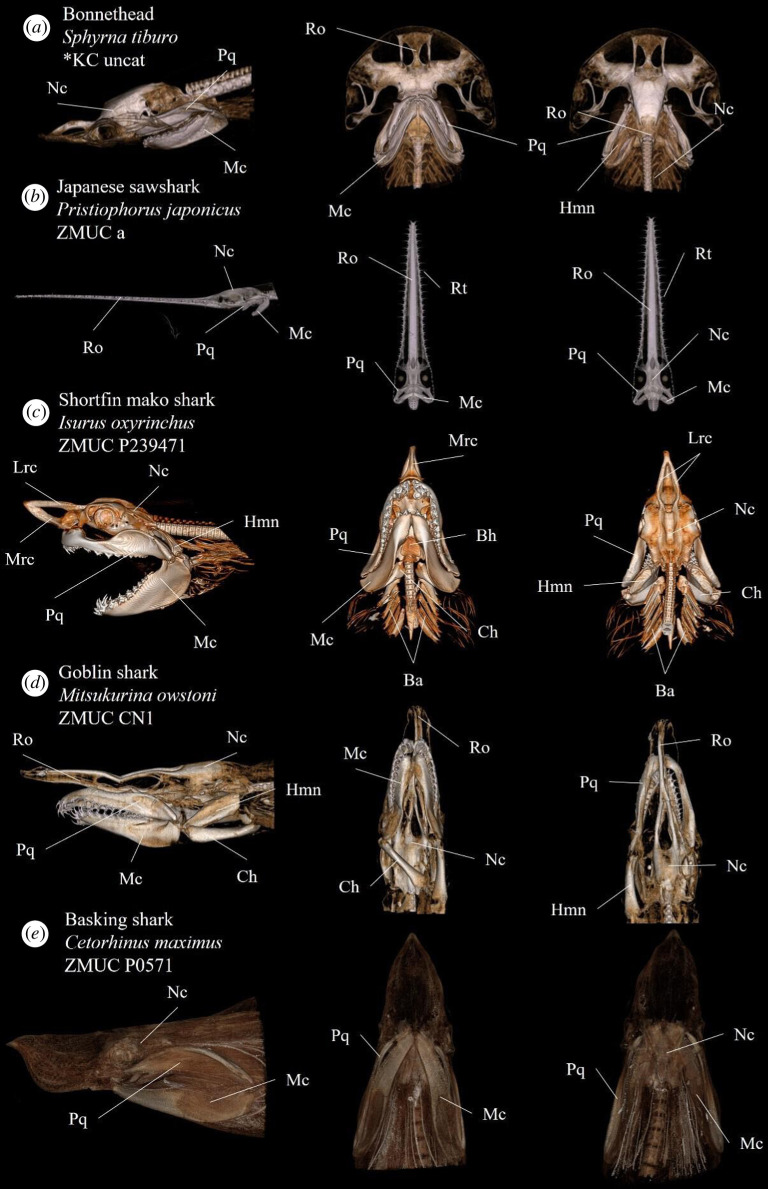
Annotated volume renderings depicting morphological variation of the elasmobranch cranial anatomy in various species. Rows display different views of the same specimens. From left to right; left lateral view, ventral view and dorsal view. Anatomical abbreviations: Bh, basihyal; Ch, ceratohyal; Hmn, hyomandibula; Lrc, Lateral rostral cartilage; Mc, Meckel’s cartilage; Mrc, Medial rostral cartilage; Nc, neurocranium; Pg, Pectoral girdle; Pq, palatoquadrate; Ro, Rostrum; Rt, Rostral teeth.

**Figure 7.**
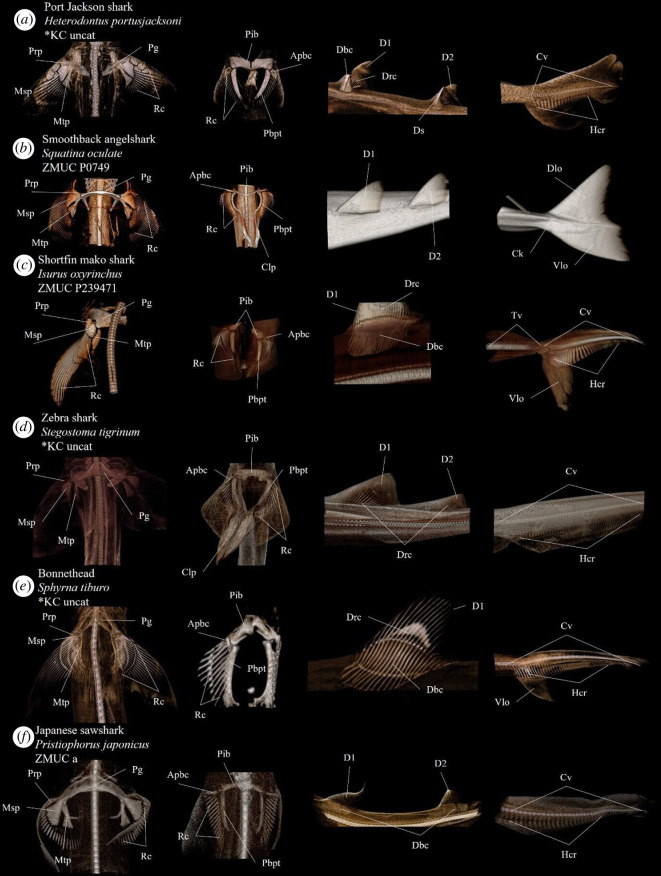
Annotated volume renderings depicting morphological variation of the elasmobranch post-cranial anatomy in various species. Anatomical abbreviations: Af, anal fin; Apbc, anterior pelvic basal cartilage; Ck, caudal keel; Cv, caudal vertebrae; Clp, claspers; D1, first dorsal fin; D2, second dorsal fin; Dbc, dorsal basal cartilage; Dlo, dorsal lobe; Drc, dorsal radial cartilage; Hcr, hypocaudal rays; Msp, mesopterygium; Mtp, metapterygium; Pbc, pectoral basal cartilage; Pbpt, pelvic basipterygium; Pg, pectoral girdle; Pib, pupoischiatic bar; Prp, propterygium; Rc, radial cartilage; Tv, trunk vertebrae; Vlo, ventral lobe.

The well-mineralized endoskeleton of the sand tiger shark enables the identification and subsequent segmentation of skeletal structures from CT scans (figure 1). Given that the imaging was performed using a clinical CT system, intended for specimens roughly the size of an adult human, the sand tiger shark is a close to optimal fit for digitizing an adult, mid-size shark in its entirety (figure 2*a*). This close fit allows for a higher image resolution, given the number of voxels comprising the specimen. To illustrate this, the total number of voxels has been calculated for a number of other shark species previously scanned using the same CT system (figure 2*b*). Additionally, the total volume (cm^3^) and length indicated as total length (TL) are also provided (figure 2*a*). Given that all specimens have been scanned using a conventional clinical CT system, the optimal size of the specimen in question is naturally also an important factor for achieving as high as possible image resolution for capturing details in the anatomy. From this, it is apparent that the number of voxels comprising the sand tiger shark specimen is much higher than for the other sharks included here (figure 2*b*). The high degree of mineralization of the endoskeleton of the sand tiger shark allows for the identification and subsequent segmentation of skeletal structures from CT scans. This, along with the high image resolution and overall post-cranial morphology, makes the sand tiger shark a strong candidate for skeletal segmentation and model construction exercises. Given the size and subsequent image resolution, the interactive model (figures 3 and 4 and electronic supplementary material, supplementary material 1–3) is able to capture numerous detailed features of individual components which can be identified and visualized in great detail. The ability to select and deselect which structures to display in the model at any given time allows one to acquire detailed three-dimensional representations of single endoskeletal structures (figures 3 and 4 and electronic supplementary material, supplementary material 1–3), while also allowing for visualizing of both positions in the body and orientation in relation to closely associated structures. The various colorations of the individual structures allow for clear, detailed distinctions of closely associated structures to be made (figures 3 and 4 and electronic supplementary material, supplementary material 1–3). Morphological variation in the cranial region of sharks makes generalizations difficult, and as such the generated model cannot capture the variation sufficiently for all sharks. For this a series of volume-renderings of additionally scanned specimens are presented in figures 5–7, showcasing the morphological variation in the cranial region as well as a selected part of the post-cranial morphology in representatives of most of the established orders of sharks as well as some of the more distinct morphologies.

## 4. Discussion

The overall size of the sand tiger shark is well suited for a clinical CT system resulting in a high image resolution of a mid-size, fully grown shark, compared with other specimens scanned here. Smaller species such as the epaulette shark (*Hemiscyllium ocellatum*) could also have been chosen and scanned using a micro-CT system due to its highly mineralized skeleton. However, in order to achieve similar image resolution, an isotropic voxel size of 90 µm would be required in order to achieve a similar voxel count as for the sand tiger shark presented here (figure 2*b*). This is not impossible; however, it is challenging to find micro-CT systems capable of fitting an epaulette shark specimen of this length (total length = 67.5 cm).

Because of the high image resolution, the constructed model is able to capture, visualize and discern more detailed features of individual three-dimensional structures (figure 4). Our presented model offers a higher degree of separation and detailed annotation of individual anatomical structures than other currently available resources such as the otherwise impressive online initiative ‘Chondrichthyan Tree of Life’ [38]. From the figures (figures 5 and 6), the variation in the orientation and elongation of the mandibular arch and the shape of the neurocranium and rostrum is apparent. These features are often closely related to feeding behaviour, sensory systems and other important traits [27,28], making generalizations regarding general morphology difficult.

The ability to scan specimens, perform digital dissections and document them in three dimensions enables scientists to create permanent digital records of animals and to make them accessible worldwide in a matter of minutes [7]. An interactive three-dimensional atlas allows for the visualization of internal morphological features while decreasing the need for invasive sampling of physical specimens and subsequent destructive dissections. For a species with a substantial size like an adult sand tiger shark, housing and curating the specimen in a natural history collection in its entirety is, in most cases, not feasible. By creating accessible, permanent digital records of species and preserving them in their entirety, accessibility increases dramatically reaching a global community [39].

One great advantage of three-dimensional models compared with physical specimens is their application in several educational formats such as larger lectures, exhibitions and as remotely accessed online material. The model offers great advantages over two-dimensional illustrations in its ability to capture orientation and allow for interactive exploration of single anatomical components, as well as larger coherent structures.

## 5. Conclusion

Given the relatively large degree of mineralization of the endoskeleton, along with the overall size, the sand tiger shark is an ideal candidate for skeletal segmentation and construction of a skeletal atlas. Given its post-cranial body morphology, the sand tiger shark is a fitting species for a more generalized shark skeletal anatomy for future educational purposes. The size of the sand tiger shark, compared with other well-mineralized shark species, makes it a well-suited candidate for a clinical CT system resulting in a high voxel resolution allowing for detailed features of the various skeletal structures to be captured and visualized in the three-dimensional model in great detail. The generation of a three-dimensional model provides remote access and insight into detailed anatomy, previously only accessible through destructive dissections of physical specimens.

## Data Availability

Raw CT data of included specimens is available on MorphoSource [40]. Individual mesh files for the segmented model are also available in this MorphoSource project. Browser-based, simplified interactive models available at SketchFab: https://sketchfab.com/CMLAUH/collections/sand-tiger-shark-skeleton-models-c6bdaa95a36842b6b7d45c8c6f3bc0da. Electronic supplementary material is available online [41].
